# Implementing State action plans for the prevention of snakebite envenoming

**DOI:** 10.1371/journal.pntd.0013157

**Published:** 2025-07-09

**Authors:** Jaideep C. Menon, Aravind M S

**Affiliations:** 1 Adult Cardiology & Public health, Amrita Institute of Medical Sciences, Kochi, Kerala, India; 2 Department of Public Health, Amrita Institute of Medical Sciences, Kochi, Kerala, India; Universidad de Costa Rica, COSTA RICA

I read with interest the letter to the editor written by Akhilesh Kumar and Maya Gopalakrishnan, wherein the authors touch on a number of aspects related to snakebite and envenoming in particular, which are co-contributory to relegating snakebite into the annals of neglected diseases. At the outset I should thank them for their interest in this much neglected disease.

In the context of a hypothesis evolving to a theory and further onto becoming a law, the level of evidence generated is what decides this transition. The ICMR (Indian Council of Medical Research) as the apex body for the formulation, coordination and promotion of biomedical research in the country is tasked with generating the necessary evidence for disease prevention, therapy and nurturing good health of the citizens [1]. Given the necessary evidence it is the Ministry of Health and Family welfare (MoHFW) that is responsible for promulgating laws and policies towards ensuring a healthier population. The CCoE (Collaborating Centre of Excellence) at AIMS (Amrita Institute of Medical Sciences), Kochi functions under the ambit of the ICMR as a collaborating centre to generate evidence in the area of snakebite and snakebite envenoming (SBE) [2].

There is evidence available to indicate geographical variability of venoms intraspecies in biochemical and toxicity profile, differential neutralization patterns to the same species across different geographies for the same commercially available anti-snake venom, morbidity and mortality reported from non-Big4 species, and the significant economic burden resulting from SBE in rural populations [3–11]. There have been questions raised regarding the quality of venom, milking techniques used, and of the efficacy of the commercially available anti-snake venom (ASV) [12–14]. All these aspects alongside the purification of venom, manufacture of ASV, testing batches of ASV for efficacy, and the effects of climate change and circumstances leading to bite (factoring for effects of rodenticides and modern agricultural practices like use of combined harvesters etc) needs to be researched [15,16]. The focus of research and publications in India has mostly been in the areas of the clinical aspects of SBE and on venom, and not as much on snakes. Much needs to be understood with regards to habitat, forage area, feeding habits, behavior, gender differences and age, and its role in snake-human conflict. The phenotypes and species of especially, the venomous snakes need to be researched in more detail and the downstream effects on SBE needs to be assessed [13,16,17]. Impoverishment and inequity are associated with snakebite in more ways than one. Certain behavioral habits associated with impoverishment like open defaecation, sleeping on the ground, not using protective covered footwear etc puts one at risk of snake-human conflict. It is estimated that 65–70% of snakebites occur in individuals between the ages of 20–65, the so called economically productive age-group. The loss of the sole earning member of a family secondary to SBE has enormous social and economic ramifications. Research on all these aspects would be beyond the ambit of any single organization in the country and would require collaboration between the different researchers cutting across specialties, working in the domain [16].

For India to be able to meet the WHO SDG (World Health Organisation-Sustainable Development Goals) 2030 the policy bodies like the NITI Aayog (which stands for National Institution for Transforming India, is an advisory body that replaced the Planning Commission, focusing on policy advisory and promoting cooperative federalism rather than financial allocation) needs to bring the different departments concerned with the issue of snakebite and SBE namely Health, Animal Husbandry, Forests, Chemicals and fertilizers, Environment and climate change, Agriculture, disaster management, irrigation and waterways, on board with a composite plan towards prevention and mitigation [16].

Dashboards and hot-spot mapping alongside digital applications facilitating identification, therapy, and transport of victims are surely a way forward given the ubiquity of a handheld device, and successful use in surveillance, monitoring and treatment of non-communicable diseases (NDC) under the NP-NCD (National Program for the Control and Prevention of complications of NCDs) [16,18].

Deaths from SBE mirror impoverishment, inequity, inadequacy of the health-system and infrastructure more than anything else. Kerala in the southern part of the country boasts of excellent healthcare facilities in both the public and private sectors. Kerala reported 34 deaths due to SBE in 2023–24, as compared to 800–900 in Odisha and over 2000 in the populous state of Uttar Pradesh [9,19,20]. The drop in mortality could be ascribed to a few factors together namely; a system of trained community volunteers trained by the forest department of the state; training of frontline health worker (ASHAs) on the nuances of first-aid, prevention and sentinel signs and symptoms; a functional network of ambulance services round-the-clock; and Apps (Sarpa and Snakepedia) which have facilitated treatment by listing hospitals in the vicinity with stocks of ASV and facilities to treat SBE [21,22]. Targeting ‘Zero snakebite deaths’ by 2030 the CCoE at AIMS organized a stakeholder meeting on the 13^th^ February, 2025 with representatives from the State health department, Forest department, ICMR institutes, NGOS, App developers, herpetologists, clinicians from both the public and private sectors and media [23]. A document statement towards a state action plan for the prevention of snakebite is being compiled along with sharing of responsibilities amongst the various stakeholders. The same initiative would be extended to the states of Maharashtra, Odisha, Himachal Pradesh and Karnataka in that order over the next 6 months.

In summary the health of the state’s citizens is the responsibility of the state and not as much the centre in the federal system of governance that India follows. For improving awareness, empowering communities especially in the so-called snakebite hotspots, a bottoms-up approach is what is advocated. While other aspects as with regards to making snakebite notifiable, quantum of compensation paid in case of death or envenoming, inclusion in the free health schemes for those below-the-poverty-line (BPL), zonal venom pools, availability of venom for research purposes, improving the quality of ASV, extending ASV cover to non-Big4 species, and the medicolegal aspects of snakebite would require a top-down approach.

The three major transition points as far as snakebite research is concerned for India are, firstly when it transited to NCDs from the miscellaneous section where it languished for decades which ensured funds for research, presently snakebite has been included under the one-health umbrella; secondly when SBE was relisted as a NTD in 2017 and thirdly snakebite being made notifiable by the MoHFW.

We believe the CCoE would help foster research, generate evidence and serve as a data-resource. The CCoE was never contemplated as a one-stop shop for SBE and could actually work as a liaison between different research groups and the ICMR and other central organizations. The National program on snakebite is being twinned with the successful one-health program on Rabies even though the contexts stand different given the availability of an anti-rabies vaccine. For India to be able to meet the WHO SDG goals 2030, a series of immediate measures need be made post-haste, involving the centre and the states, policy makers and the community, a contextualized top-down and bottoms-up approach, and strengthening of the national program with adequate human resources and the finances. The four pillars provided by the WHO document provide a broad framework to address this most neglected of NTDs. Each of the states of the country need bring a contextual State Action Plan for Prevention of SBE building on the NAP-SE and WHO-SDG frameworks for implementation in the respective states. The Union and the States need consider this is a health priority given that snakebite is both eminently preventable and treatable.
